# Control of breast cancer patient-derived xenografts by CD70-targeted HIT-CAR T cells

**DOI:** 10.3389/fimmu.2026.1852493

**Published:** 2026-08-26

**Authors:** Pieter L. Lindenbergh, Vinagolu K. Rajasekhar, Irina Linkov, Jorge Mansilla-Soto, Sophie A. Hanina, Michel Sadelain

**Affiliations:** 1 Columbia Institute for Cell Engineering and Therapy (CICET), Vagelos College of Physicians and Surgeons, Columbia University Irving Medical Center, New York, NY, United States; 2 Department of Hematology, Cancer Center Amsterdam, Amsterdam UMC, Amsterdam, Netherlands; 3 Orthopedic Service, Department of Surgery, Memorial Sloan Kettering Cancer Center, New York, NY, United States; 4 Pathology Core Facility, Memorial Sloan Kettering Cancer Center, New York, NY, United States; 5 Departments of Immunology, Bioengineering, and Blood & Marrow Transplant and Cellular Immunotherapy, H. Lee Moffitt Cancer Center & Research Institute, Tampa, FL, United States

**Keywords:** breast cancer, CAR T cells, CD70, immunotherapy, solid tumor

## Abstract

CAR T cell therapies have shown clinical success in several tumor types, though not in breast cancer, for which advanced stages remain incurable. CD70 is expressed in a broad range of solid tumor types and is emerging as a molecular target for CAR T cells in renal and ovarian malignancies. Here we show that a subset of breast cancer cell lines and patient-derived xenografts express CD70, albeit to varying levels. Leveraging a recently developed HLA-independent T cell (HIT) receptor with greater sensitivity to CD70 than conventional CAR designs, we assessed whether CD70 can be targeted to eliminate breast cancer cells. We observed that CD70-targeted HIT T cells showed antigen-specific activation *in vitro*, and cytotoxicity against breast cancer cell lines and patient-derived xenograft cells across a broad range of CD70 levels. Implanted in the murine mammary fat pad, high CD70-expressing patient-derived xenografts were readily eliminated by both conventional CAR and HIT T cells. Against xenografts with lower CD70 expression, however, CD70-targeted HIT T cells engineered with supplemental costimulation outperformed conventional CAR T cells by inhibiting tumor outgrowth in mice, and conferred a significant survival benefit. These findings establish that CD70 is a targetable antigen in breast cancer and highlight the potential of enhanced-sensitivity receptor designs such as HIT receptors to engage a broader range of tumors.

## Introduction

Breast cancer remains a leading cause of cancer death in women, highlighting the unmet need for curative treatments (1, 2). T cell therapies and immune checkpoint inhibition targeting the CTLA4 and PD-1 axes have shown durable remissions in several malignancies including leukemia, multiple myeloma, melanoma and lung cancer (3–5). However, current immune checkpoint inhibition regimens have only improved survival in a subset of breast cancer patients (6, 7), and are only approved for metastatic, PD-L1^+^, triple-negative (ER^-^PR^-^HER2^-^) breast cancer (8). Multiple clinical studies indicate that immune checkpoint inhibition in breast cancer requires a combination with chemotherapy (5–10), which induces immunogenic cell death and increases tumor antigen presentation to T cells (11–16). This suggests that TCR-engagement is a limiting factor in eliciting clinically meaningful T cell responses against breast tumors. The genetic engineering of T cells to express Chimeric Antigen Receptors (CAR) circumvents the requirement for naturally occurring tumor-reactive TCRs by targeting the T cells to any cell surface antigen. CAR T cells for breast cancer have been developed against a range of molecular targets, including HER2 (17, 18), EGFR (19), mesothelin (20, 21) and B7-H3 (22), and trials targeting these molecules in breast cancer patients are ongoing (8, 23).

CD70 has also been proposed as a target for CAR T cells for breast cancer (8, 24). Immuno-histochemistry studies by two independent groups reported CD70 detection in 47.6% (25) and 48.4% (26) of breast cancer cases including primary and metastatic tumors. Furthermore, higher RNA levels of CD70 are associated with a more aggressive, basal-like phenotype of breast cancer (27). Here, we set out to assess whether CD70 detection in clinical breast cancer samples may predict their sensitivity to CD70-targeted CAR T cell therapy. To evaluate the range of targetable CD70 levels, we included a conventional second-generation CAR and an HLA-independent TCR (HIT). In HIT receptors, scFv heavy and light chains are fused to the TCR constant β and α domains, conferring greater antigen-sensitivity than conventional CAR designs and enabling the targeting of lowly expressed antigens (28, 29). We furthermore assessed the effect of cotransduction with costimulatory molecules CD80 and 4-1BBL, which has been shown to improve the *in vivo* efficacy of HIT T cells (28, 29), but not conventional, second-generation CAR T cells (29, 30). We found that CD70-targeted HIT (70HIT) T cells can control breast cancer cells *in vitro* and in orthotopic patient-derived xenograft (PDX) models.

## Methods

### T cells and engineering

**Figure 1:**
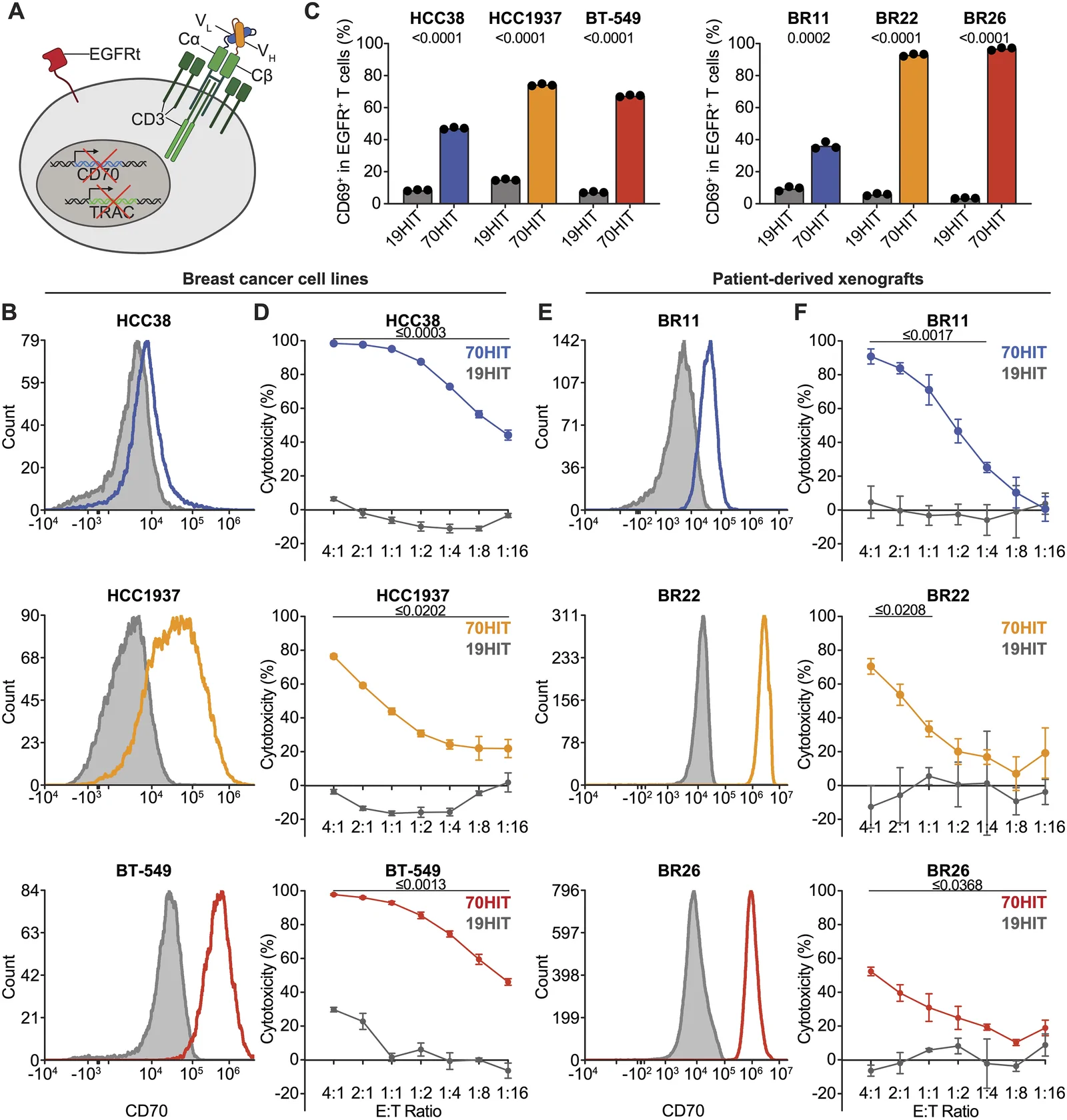
*In vitro* cytotoxicity of CD70-targeted HIT T cells against breast cancer cells. **(A)** Schematic depiction of CD70^KO^TRAC^KO^ T cell transduced with 70HIT-EGFRt. **(B)** CD70 expression on cell lines HCC38, HCC1937 and BT-549, gated on live singlets. Fluorescence Minus One controls shown in grey. **(C)** Fraction of CD69^+^ 19HIT or 70HIT T cells upon co-culture with tumor cell lines (left) or PDX cells (right) for 20 hours (n=3), gated on EGFRt^+^ T cells. **(D)** Cytotoxicity of 19HIT and 70HIT T cells against tumor cell lines at indicated Effector to Target (E:T) ratios at 18 hours (n=3). **(E)** CD70 expression on BR11, BR22 and BR26 PDX cells, gated on live singlets. Fluorescence Minus One controls shown in grey. **(F)** Cytotoxicity of 19HIT and 70HIT T cells against PDX cells at 18 hours (n=3-4). Cytotoxicity results against BR11 and BR26 **(F)** are representative of three experiments; other data from single experiments. Image created with BioRender.com **(A)**. P-values: unpaired, two-tailed Welch’s t test **(C)**; unpaired, two-tailed Welch’s t test with two-stage step-up method of Benjamini, Krieger and Yekutieli **(D, F)**.

**Figure 2:**
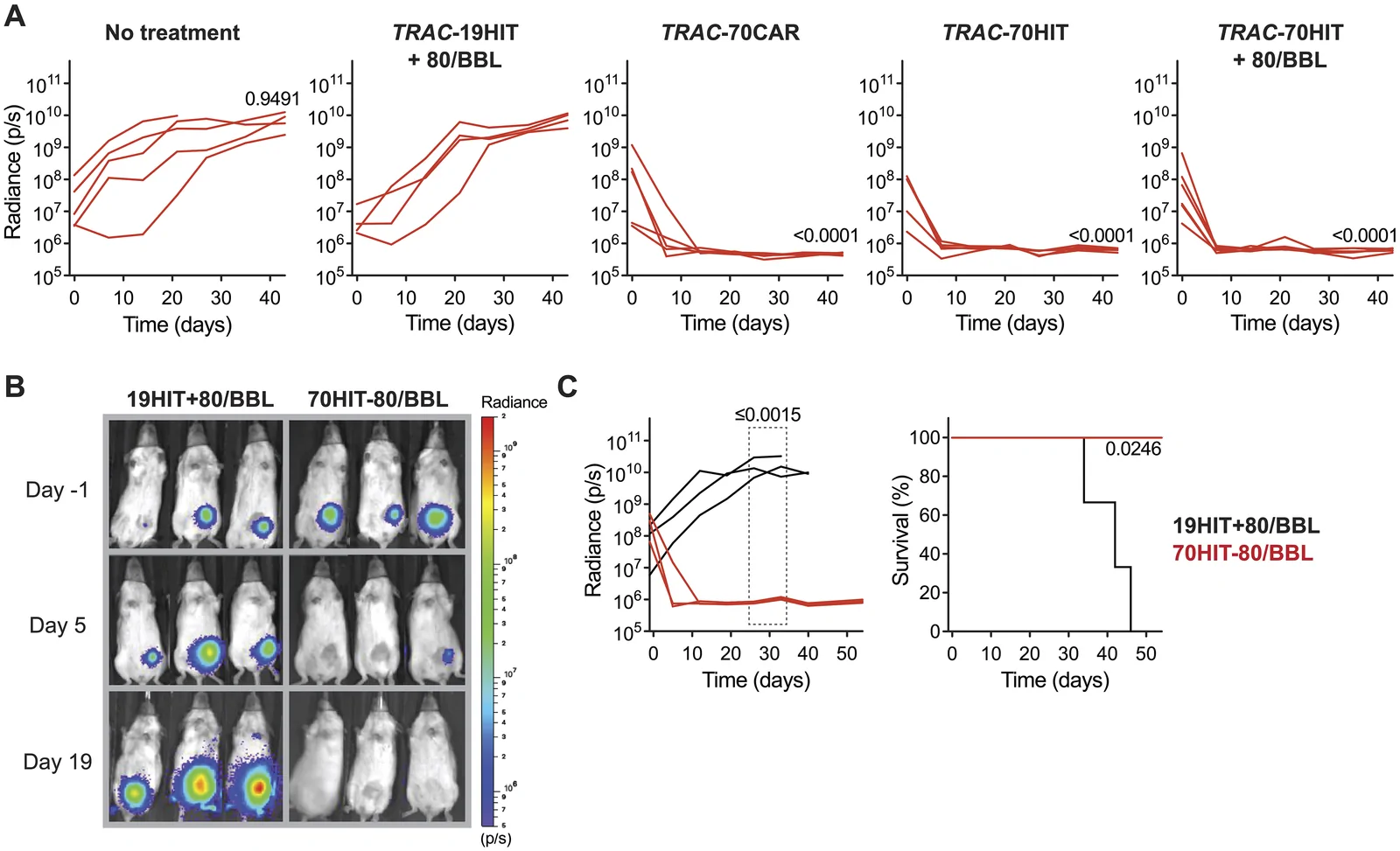
Elimination of an orthotopic MFI^high^ PDX by CD70-targeted CAR and HIT T cells. **(A)** Tumor burden of female NSG mice that were orthotopically implanted with 1×10^6^ BR26 cells 6 days prior to intravenous injection of 3×10^6^ CAR T cells (n=4-6). 19HIT, 70CAR and 70HIT genes were expressed from the *TRAC* locus using CRISPR/Cas9 and AAV, and 80/BBL was delivered through additional retroviral transduction. **(B)** Bioluminescent tumor signal overlaid with pictures, **(C)** tumor burden and survival of mice that were implanted with 2×10^6^ BR26 cells 7 days prior to injection of 2×10^6^ retrovirally transduced CAR T cells. 19HIT + 80/BBL T cells were cotransduced with 19HIT and 80/BBL retroviral vectors (n=3). 70HIT-80/BBL T cells were engineered with a single retroviral vector encoding 70HIT, CD80 and 4-1BBL (n=3). Data from single experiments. P-values: two-way ANOVA with Sidak’s test comparing all condition(s) against 19HIT + 80/BBL, showing result for Day 43 **(A)** or Day 26 and 33 **(C)**; log-rank test **(C)**.

**Figure 3:**
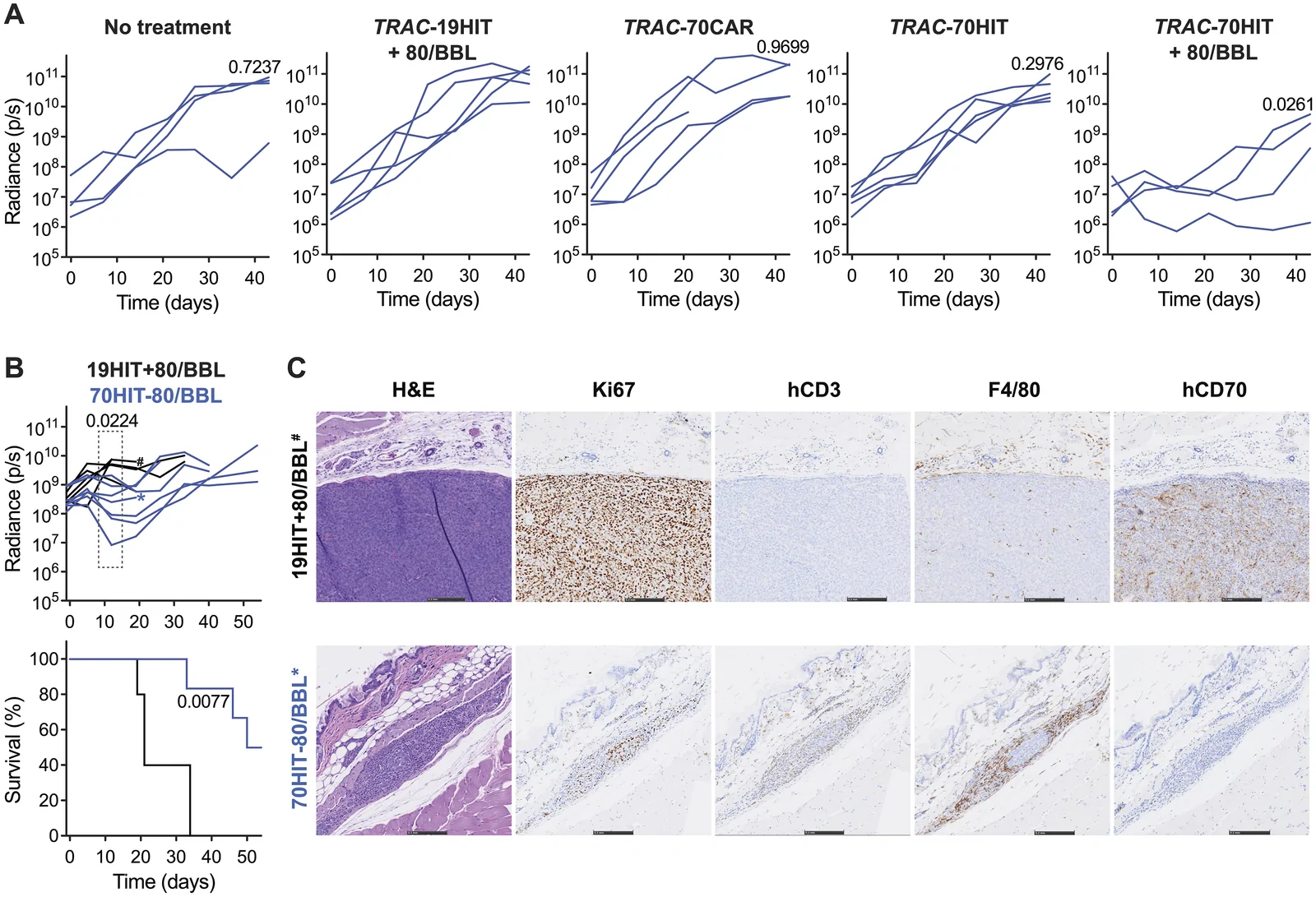
Rejection of CD70^+^ tumor cells from MFI^low^ PDX by CD70-targeted HIT T cells. **(A)** Tumor burden of mice that were implanted with 1×10^6^ BR11 cells 6 days prior to injection of 3×10^6^ CAR T cells (n=4-5). 19HIT, 70CAR and 70HIT genes were expressed from the *TRAC* locus using CRISPR/Cas9 and AAV, and 80/BBL was delivered through additional retroviral transduction. **(B)** Tumor burden and survival of mice that were implanted with 3×10^6^ BR11 cells 7 days prior to injection of 2×10^6^ retrovirally transduced CAR T cells. 19HIT + 80/BBL T cells were cotransduced with 19HIT and 80/BBL retroviral vectors (n=5). 70HIT-80/BBL T cells were engineered with a single retroviral vector encoding 70HIT, CD80 and 4-1BBL (n=6-7; survival analysis does not include animal indicated with *). **(C)** Microscopic images of tumors of mice indicated with # and * in **(B)**. Tumor sections were H&E stained, or immunohistochemically stained for Ki67, human CD3 (hCD3), F4/80, and hCD70. Black bars represent 0.2 mm. Data from single experiments. P-values: two-way ANOVA with Sidak’s test comparing all condition(s) against 19HIT + 80/BBL, showing result for Day 43 **(A)** or Day 12 **(B)**; log-rank test **(B)**.

Human peripheral blood mononuclear cells from healthy donors were acquired from the New York Blood Center (Institutional Review Board-exempted). All blood products were handled following the required safety and ethical procedures. T cells were purified using negative magnetic isolation (Pan T Cell Isolation Kit, Miltenyi Biotec). T cells were activated with CTS Dynabeads CD3/CD28 (ThermoFisher) and cultured in XVIVO-15 (Lonza) supplemented with 5% Human Serum AB (Gemini), 50 U/mL Penicillin-Streptomycin (Gibco), 5 ng/mL human interleukin-7 and 5 ng/mL human interleukin-15 (Miltenyi Biotec). Two days after bead activation, *CD70* and *TRAC* were gene-edited with CRISPR/Cas9 as described previously (28), using gRNA containing the following spacer sequences: 5’-GGGCUUGGUGAUCUGCCUCG for *CD70* and 5’-CAGGGUUCUGGAUAUCUGU for *TRAC*. Next, T cells were retrovirally transduced in plates coated with Retronectin (Takara) to deliver constructs expressing both chains of 19HIT or 70HIT and EGFRt (**Figure 1**), and, if indicated, were co-transduced with CD80 and 4-1BBL (80/BBL) (**Figures 2B, C**, **3B, C**), or were transduced with a construct expressing both chains of 70HIT, CD80 and 4-1BBL (**Figures 2B, C**, **3B, C**; **Supplementary Figure 5**), which were cloned into the SFG γ-retroviral vector (31) combining previously used sequences (28, 29) and using standard molecular biology techniques. To achieve similar expression levels between the compared constructs, the experiments shown in **Figures 2A**, **3A** were conducted with T cells that underwent Adeno-associated viruses (AAV) mediated knock-in of second-generation CD70-28z-1XX CAR, 70HIT or 19HIT genes into the *TRAC* locus and, if indicated, subsequent retroviral transduction with CD80 and 4-1BBL as described previously (28, 29). Ablation of CD70 and TRAC (>80%) and transduction were confirmed using flow cytometry and cells were not sorted prior to their use in assays. The stated numbers of used T cells represent the numbers of transduced T cells.

### Tumor cell lines

The triple-negative ductal carcinoma cell lines HCC38, HCC1937 and BT-549 were purchased from ATCC (CRL-2314, CRL-2336, HTB-122) and cultured in RPMI-1640 (Corning) supplemented with 10% FBS (Neuromics), 50 U/mL Penicillin-Streptomycin. Culture medium of BT-549 was also supplemented with Insulin-Transferrin-Selenium (ITS, Corning). Cell lines were retrovirally transduced to co-express firefly luciferase (FFLuc)-green fluorescent protein (GFP) and sorted for GFP.

### Patient-derived xenograft lines

PDX lines were derived from bone metastases of breast cancer patients aged 39-87 (**Supplementary Table 2**) with informed consent (Institutional Review Board protocols 97-094, 12-245, and 06-107), transduced and maintained as described previously (32, 33). The PDX cells were transduced with a third-generation lentiviral vector derived from a pUltra-Chili-FFLuc plasmid (Addgene, 48688) encoding dTomato and firefly luciferase and maintained in RMPI supplemented with 3% FBS, 50 U/ml Penicillin-Streptomycin (Gibco) and 2mM L-Glutamine (Gibco).

### Flow cytometry

T cells were stained with AF647-conjugated anti-human EGFR (Hu1, R&D Systems, FAB9577R), PE-conjugated anti-human CD70 (Ki-24, BD Pharmingen, 555835) and eFluor506 fixable viability dye (eBioscience). To assess activation induced CD69 expression, HIT T cells were co-cultured with tumor cells in a 2:1 ratio for 20 hours, and stained with eFluor506 fixable viability dye, AF647-conjugated anti-human EGFR and APC-R700-conjugated anti-human CD69 (FN50, BD Biosciences, 565155). To assess the pre-infusion phenotype, cryopreserved T cells were thawed and stained with AF647-conjugated anti-human EGFR, BB515-conjugated anti-human CD25 (2A3, BD Biosciences, 564467), PE-conjugated anti-human CD69 (FN50, Biolegend, 310905), BUV737-conjugated anti-human PD-1 (EH12.1, BD Biosciences, 612791), BV785-conjugated anti-human TIM-3 (F38-2E2, Biolegend, 345032), PerCP-eFlour710-conjugated anti-human LAG-3 (3DS223H, eBioscience, 46-2239-42), PE-Cyanine7-conjugated anti-human TCRαβab (IP26, eBioscience, 25-9986-42) and eFluor506 fixable viability dye.

Single cell suspensions of cell lines and PDX cells were incubated with Human TruStain FcX (Biolegend) and stained with PE- or BUV737-conjugated anti-human CD70 (Ki-24, BD Horizon, 555835 or 612856) or BV421-conjugated anti-human PD-L1 (29E.2A3, BioLegend, 329713) and eFluor506 fixable viability dye. Measured CD70 levels in tumor cells were classified as low, medium or high based on the ratio between the Median Fluorescence Intensity (MFI) of anti-CD70 stained and unstained cells (Fluorescence Minus One controls). We considered a ratio below 12 low (MFI^low^), a ratio between 12 and 120 medium (MFI^medium^) and a ratio above 120 high (MFI^high^). To estimate the number of CD70 molecules per cell, Antibody Binding Capacity (ABC) was measured on PE-conjugated anti-human CD70-stained cell lines with the Quantibrite Beads (PE Fluorescence Quantitation Kit, BD Biosciences), using cells incubated with a PE-conjugated isotype control (J606, BD Pharmingen, 556659) to subtract nonspecific ABC. ABC was measured on BUV737-conjugated anti-human CD70-stained PDX cells with the Quantum Simply Cellular kit (Bangs Laboratories), using cells incubated with a BUV737-conjugated isotype control (J606, BD Horizon, 612854) to subtract nonspecific ABC.

### *In vitro* cytotoxicity assays

Tumor cells expressing FFLuc were cocultured at the indicated Effector to Target (E:T) ratios. D-Luciferin (eLUCK, GoldBio) was added to each well after 18 hours and photon emission was detected in a luminescence plate reader. The assays were run in replicates (n=3–4 wells per ratio and condition) and the lysis was calculated relative to the signal in live control wells (no effectors added).

### Mouse models

The indicated number of BR26 and BR11 PDX cells mixed with Matrigel Matrix (Corning, 354234) were implanted in the mammary fat pad of 6–8 weeks old female NOD.Cg-*Prkdc^scid^ Il2rg^tm1Wjl^*/SzJ (NSG) mice (The Jackson Laboratory, 005557). After 6 or 7 days the indicated number of CAR T cells was injected in the tail vein (Day 0 was defined as the day of treatment). Tumor burdens were monitored by bioluminescence imaging using the IVIS Imaging System and Living Image software (Revvity). Mice were euthanized if the tumor reached a diameter larger than 2 centimeter or if their condition reached a humane endpoint (impaired locomotion, hunched posture or lethargy).

### Immunohistochemistry

Tumor tissue was collected from the mice indicated with # and * in **Figure 3B** 21 days after treatment. Mouse # was treated with 19HIT + 80/BBL, had reached an endpoint and was included in the survival analysis. Mouse * was selected for sample collection from the animals treated with 70HIT-80/BBL, had not reached an endpoint on day 21 and was not included in the survival analysis. Formalin fixed paraffin embedded tumor tissue sections were immunohistochemically stained as described previously (29). CD70 staining was done using an EDTA pH9 retrieval solution and clone 301731 (R&D Systems, MAB2738) at a 1:100 dilution. Ki67 staining was done using an EDTA pH9 retrieval solution and clone D3B5 (Cell Signaling Technology, 12202) at a 1:500 dilution. F4/80 staining was done using a citrate pH6 retrieval solution and clone BM8 (Invitrogen, 14-4801-85) at a 1:100 dilution. Human CD3 staining was done using a citrate pH6 retrieval solution and clone MRQ-39 (LSBio, C202826) at a 1:500 dilution. Standard H&E staining was performed.

## Results

### CD70-targeted HIT T cells mediate *in vitro* cytotoxicity against breast cancer cells

Prompted by the reported (34, 35) detection of CD70 RNA (**Supplementary Figure 1**) and protein in clinical breast cancer samples (25, 26), we set out to assess CD70 as a molecular target for CAR T cell therapy in breast cancer. We transduced healthy donor T cells to express a 70HIT receptor (29). Because T cells may express CD70 (36, 37), we ablated CD70 expression in the T cells to avoid fratricide, and we mitigated alloreactivity by targeting the *TRAC* locus as previously described (29, 38) (**Figure 1A**). Flow cytometric analysis of the T cells confirmed expression of the construct and effective ablation of CD70 in 70HIT T cells (**Supplementary Figure 2**). CD19-targeted HIT (19HIT) T cells served as controls, as CD19 is not expressed on breast cancer cells. Next, we measured the MFI of anti-CD70 staining (**Figure 1B**) and quantified CD70 molecules (**Supplementary Table 1**) on commonly used triple-negative breast cancer cell lines and found that HCC38 and HCC1937 had a low MFI (ratio stained:unstained < 12) and that BT-549 was MFI^medium^ (12 < ratio < 120). We observed that the T cell activation marker CD69 was strongly induced on 70HIT T cells upon co-culture with HCC38, HCC1937 and BT-549 in contrast to control 19HIT T cells (**Figure 1C**). Consistently, we observed antigen-specific killing of these three cell lines in an 18-hour cytotoxicity assay (**Figure 1D**).

To corroborate that CD70 is a target for HIT T cells, we screened six PDXs derived from metastatic breast tumors for CD70 expression (**Supplementary Table 2**). We found that three PDXs had a high MFI for CD70 (ratio stained:unstained > 120) and that three had a low MFI (ratio <12), showing that CD70 is present across a subset of patient-derived hormone receptor positive and triple-negative cancer cells (**Figure 1E**; **Supplementary Table 2**). CD69 expression was induced on 70HIT T cells following co-culture with MFI^high^ (BR22 and BR26) and MFI^low^ (BR11) PDX cells, unlike on control 19HIT T cells (**Figure 1C**). Consistently, 70HIT T cells were able to kill both MFI^high^ and MFI^low^ PDX cells in a dose-dependent manner, while control 19HIT T cells showed no toxicity (**Figure 1F**). CD70 expression levels did not strictly correlate with 70HIT T cell cytotoxicity, suggesting a role for factors beyond target antigen density, including molecules known to modulate CAR T cell cytotoxicity such as PD-L1 (**Supplementary Figure 3**) (39, 40). Altogether, these findings demonstrate that 70HIT T cells are cytotoxic against CD70-expressing breast cancer cells across both established cell lines and PDX cells *in vitro*. Next, we assessed the *in vivo* efficacy of 70HIT T cells against representative MFI^high^ or MFI^low^ PDXs.

### CD70-targeted conventional CAR and HIT T cells both eliminate MFI^high^ PDX

To assess the potential of CD70-targeted CAR T cells against MFI^high^ PDXs *in vivo*, we implanted 1×10^6^ BR26 cells in the mammary fat pad of female NSG mice 6 days prior to intravenous injection of 3×10^6^ CAR T cells. To ensure comparable levels of CAR expression, all CAR constructs used in this experiment were expressed from the *TRAC*-locus through CRISPR/Cas9 and AAV mediated knock-in (29, 38). We created conventional, CD28-based, CD70-targeted CAR (*TRAC-*70CAR) T cells, *TRAC-*70HIT T cells and *TRAC-*70HIT T cells cotransduced to express costimulatory molecules CD80 and 4-1BBL (*TRAC-*70HIT+80/BBL), which were previously shown to improve the therapeutic potency of HIT T cells, unlike conventional CAR T cells which possess a built-in costimulatory domain (28–30). *TRAC-*19HIT T cells cotransduced with 80/BBL were created as control. Before infusion, all products (**Supplementary Figure 4**) expressed CD25. *TRAC*-70CAR T cells expressed higher CD69 than *TRAC*-70HIT with or without 80/BBL. TIM-3 was similar across all products, whereas PD-1 and LAG-3 were lower on *TRAC*-70HIT T cells compared to *TRAC*-70CAR and *TRAC-*70HIT+80/BBL. Treatment with *TRAC-*19HIT+80/BBL T cells did not impact on BR26 PDX outgrowth. BR26 PDXs were completely and durably eliminated by *TRAC-*70HIT T cells as well as by the less sensitive, conventional *TRAC-*70CAR T cells (**Figure 2A**), consistent with the ability of *TRAC-*70CAR T cells to target CD70 provided that its expression is homogenously high (29). Cotransduction with 80/BBL did not further enhance BR26 clearance by *TRAC-*70HIT T cells (**Figure 2A**).

In an independent experiment, we set out to test T cells retrovirally transduced with a novel, single vector encoding 70HIT, CD80 and 4-1BBL (70HIT-80/BBL; **Supplementary Figure 5**), further challenging the potency of these cells by increasing the BR26 tumor burden (implantation of 2×10^6^ cells 7 days prior to treatment) and decreasing the dose of T cells to 2×10^6^. Despite the lower effector-to-target ratio, the 70HIT-80/BBL T cells effectively eliminated BR26 PDXs, whilst 19HIT + 80/BBL T cells did not (**Figures 2B, C**). The effective clearance of BR26 PDXs resulted in long-term, tumor-free survival (**Figure 2C**).

### 70HIT+80/BBL T cells slow down growth of MFI^low^ PDX

To test CD70-targeted CAR T cells against a MFI^low^ PDX that was susceptible to *in vitro* lysis (**Figure 1F**), mice were implanted with 1×10^6^ BR11 cells and treated with 3×10^6^ *TRAC-*19HIT+80/BBL, *TRAC-*70CAR, *TRAC-*70HIT, or *TRAC-*70HIT+80/BBL T cells (**Supplementary Figure 4**) after 6 days. A modest delay in tumor outgrowth was observed in a subset of the mice treated with *TRAC-*70CAR (2 out of 5) and *TRAC-*70HIT (3 out of 5) during the first two weeks; however, all tumors subsequently progressed with kinetics comparable to untreated or *TRAC-*19HIT+80/BBL controls (**Figure 3A**). Consistent with previous reports (28–30, 41), cotransduction with 80/BBL enhanced the activity of *TRAC-*70HIT T cells, significantly delaying BR11 PDX outgrowth (**Figure 3A**).

To evaluate 70HIT-80/BBL T cells under more stringent conditions, we increased the tumor burden by implanting 3×10^6^ BR11 cells 7 days prior to treatment while reducing the T cell dose to 2×10^6^. Despite these more challenging conditions, retrovirally transduced 70HIT-80/BBL T cells significantly controlled BR11 PDX outgrowth and offered a survival benefit over the control treatment with 19HIT + 80/BBL T cells (**Figure 3B**).

Immunohistochemical analysis of tumor sections collected 21 days after treatment revealed qualitative differences between treatments. Consistent with the difference in bioluminescent tumor signal (**Figure 3B**; mice indicated with # and *), a 19HIT + 80/BBL T cell-treated mouse displayed a larger Ki67^+^ tumor mass than a 70HIT-80/BBL T cell-treated mouse (**Figure 3C**). Staining for human CD3 and the murine macrophage marker F4/80 in 19HIT + 80/BBL T cell-treated tumor showed few CAR T cells and macrophages (**Figure 3C**). In contrast, the 70HIT-80/BBL T cell-treated tumor showed increased accumulation of human T cells around and inside the tumor, arguing against their exclusion. Consistent with the ability of antigen-activated CAR T cells to recruit myeloid cells to the tumor site (42, 43), the 70HIT-80/BBL T cell-treated tumor showed a higher density of F4/80^+^ murine macrophages in the peritumor tissue. Notably, in the CD19-HIT+ 80/BBL T cell-treated tumor, CD70 staining was heterogenous, whereas CD70 staining was absent in the 70HIT-80/BBL T cell-treated tumor. Together these data indicate that 70HIT-80/BBL T cells conferred tumor control by targeting CD70-expressing breast cancer cells.

## Discussion

Our findings support that CD70 is a potential target for CAR T cell therapy in breast cancer, particularly when using a highly sensitive CAR design. Half of the PDXs screened in this study expressed high levels of CD70, consistent with reports indicating that a subset of clinical breast cancer cases is CD70^+^ (25, 26). We found that 70HIT T cells could lyse breast cancer cell lines and PDX cells *in vitro* across a broad range of CD70 MFIs (**Figure 1**). We observed that both conventional CAR and HIT T cells effectively eliminated orthotopically implanted MFI^high^ BR26 PDX (**Figure 2**). In this setting with abundant antigen, 70HIT T cells did not require 80/BBL costimulation, consistent with their rapid clearance of BR26 PDX. These observations suggest a reduced need for T cell persistence in the context of high CD70 expression, which future work could address by comparing T cell quantities between different T cell treatments in this model. Conventional CAR T cells failed to control the MFI^low^ BR11 PDX, while 70HIT T cells with 80/BBL costimulatory support achieved greater anti-tumor activity (**Figure 3A**).

Besides its role as a target antigen, high CD70 expression on tumor cells may also influence CAR T cell function through engagement of its costimulatory receptor CD27. While excessive CD70 exposure is associated with T cell exhaustion (44, 45), induces apoptosis (46, 47) and promotes immunosuppression through regulatory T cells (48) and tumor-associated macrophages (49), transient CD27 signaling provides potent costimulatory signals that enhance T cell activation, expansion, survival and memory formation (50–52). CD27 signaling has been shown to improve survival of CD70-targeted T cells (53) and to enhance the capacity of NKG2D-targeted CAR T cells to inhibit outgrowth of a breast cancer cell line *in vivo* (54). Thus, high CD70 expression on the tumor cells may not only facilitate rapid and complete tumor clearance by enabling strong CAR activity but may also enhance the T cell response by providing costimulatory signals. Supplemental costimulation was, however, required in a low-CD70 setting (**Figure 3A**), for which we streamlined vector delivery with a novel tetracistronic construct encoding both chains of 70HIT, CD80, and 4-1BBL, thereby simplifying CAR T cell manufacturing (**Figure 3B**).

Despite the elimination of CD70^+^ cells from MFI^low^ BR11 PDX, which was associated with significant survival benefit, 70HIT-80/BBL T cells did not achieve complete tumor clearance (**Figure 3B**). CD70 expression has been shown to be silenced by DNA hypermethylation of its promoter in breast cancer cell lines (55). In addition, we have shown recently that CD70 expression can be repressed through trimethylation of histone H3 on lysine 27 in renal, pancreatic and ovarian cancer, implicating histone-modifying enzymes such as EZH2 (29). If these mechanisms contribute to CD70 silencing in breast cancer, pharmacological modulation using DNA methyltransferase inhibitors, such as azacitidine, or EZH2 inhibitors, may enhance and stabilize antigen expression, thereby improving CAR T cell efficacy (29). Alternatively, 70HIT T cells could be incorporated in dual-targeting strategies, wherein their ability to target low antigen levels may reduce the likelihood of antigen escape of heterogenous tumors. Although CD70 expression is largely limited to tumor and immune cells (29), clinical translation of these findings will require careful assessment of on-target/off-tumor toxicities.

In sum, our results provide preclinical support for the inclusion of homogenously CD70^high^ breast cancers in basket trials of CD70-targeted conventional CAR T cells (56–58), and further suggest that highly sensitive CARs such as HIT receptors augmented with 80/BBL costimulation may be active against a broader range of CD70^low^ breast cancers.

## Data Availability

The raw data supporting the conclusions of this article will be made available by the authors, without undue reservation.
