# Supplementary material for: Control of breast cancer patient-derived xenografts by CD70-targeted HIT-CAR T cells
Source: Front Immunol. 2026 Aug 26;17:1852493. doi: 10.3389/fimmu.2026.1852493 (PMC13553483; doi:10.3389/fimmu.2026.1852493)
Supplement: Supplementary file 1 [file DataSheet1.docx]

Supplementary Material

**Supplementary Figure 1.** CD70 RNA abundance in breast cancer tissue (n=1108) and healthy breast tissue (n=354), combining data from The Cancer Genome Atlas (https://www.cancer.gov/tcga) and the Genotype Tissue Expression Project (GTEx Consortium, *Nature*, 2017). Figure was generated with Gepia3.bioinfoliu.com described in (34).

**Supplementary Figure 2.** Wildtype, non-transduced (NTD) T cells and TRAC^KO^CD70^KO^ T cells transduced with 19HIT-EGFRt or 70HIT-EGFRt; gated on live singlets.

**Supplementary Figure 3.** PD-L1 expression on BR11, BR22 and BR26; gated on live singlets.

**Supplementary Figure 4.** Activation and exhaustion marker expression on *TRAC*-70CAR, *TRAC*-70HIT and *TRAC*-70HIT+80/BBL T cells used in the experiments shown in Figures 2A and 3A; gated on live, single, TCRαβ^+^ or EGFRt^+^ T cells.

**Supplementary Figure 5.** Representative flow plots of CD80 and 4-1BLL expression on CD70^WT^TRAC^WT^, non-transduced T cells (Wildtype T cells) and CD70^KO^TRAC^KO^ T cells that were retrovirally transduced with a SFG γ-retroviral vector to co-express 70HIT, CD80 and 4-1BBL (70HIT-80/BBL T cells); gated on live, single, CD3^+^ T cells.

| **Cell line** | **ABC** |
| --- | --- |
| HCC38 | 235 |
| HCC1937 | 848 |
| BT-549 | 67526 |

**Supplementary Table 1.** Estimated anti-CD70 Antibody Binding Capacity (ABC) of breast cancer cell lines used in this study as determined by flow cytometry using Quantibrite Beads from BD Biosciences.

|  |  |  | **Median Fluorescence Intensity** | | |  |
| --- | --- | --- | --- | --- | --- | --- |
| **PDX** | **Diagnosis** | **Subtype** | **Unstained** | **Stained** | **Ratio** | **ABC** |
| BR07 | Invasive ductal carcinoma | ER+ | 4453 | 18827 | 4.2 |  |
| BR11 | Invasive carcinoma | ER+ | 1807 | 20637 | 11.4 | 1496 |
| BR18 | Ductal and lobular carcinoma in situ | ER-PR-HER2- | 1066 | 5290 | 5.0 |  |
| BR22 | Ductal carcinoma in situ | ER-PR-HER2- | 13464 | 2537025 | 188.4 | >69845 |
| BR23 | Invasive ductal carcinoma | ER+PR+ | 11387 | 2491624 | 218.8 |  |
| BR26 | Poorly differentiated carcinoma | ER+PR+HER2- | 6677 | 832505 | 124.7 | >69845 |

**Supplementary Table 2.** Diagnosis, subtype and CD70 expression of patient-derived xenografts screened and used in this study. PDXs were derived from bone metastases of breast cancer patients aged 39-87 and were analyzed for CD70 expression using flow cytometry to determine Median Fluorescence Intensity and estimated Antibody Binding Capacity (ABC) using a Quantum Simply Cellular kit from Bangs Laboratories.
